# Metabolic Dysfunction-Associated Steatotic Liver Disease in Children with Obesity: Sex-Stratified Analysis of Hepatic Enzyme Profiles and Serum Uric Acid

**DOI:** 10.3390/healthcare13172219

**Published:** 2025-09-04

**Authors:** Tian Zhang, Yi Qian, Jin Zhang, Naijun Wan

**Affiliations:** Department of Pediatric Internal Medicine, Beijing Jishuitan Hospital, Capital Medical University, Beijing 100035, China; tianbing1214@sina.com (T.Z.); qianyi0530@126.com (Y.Q.); zjdct@163.com (J.Z.)

**Keywords:** children, obesity, non-alcoholic fatty liver disease, metabolic dysfunction-associated steatotic liver disease, alanine aminotransferase, serum uric acid

## Abstract

**Background/Objectives:** Sex differences in metabolic dysfunction-associated steatotic liver disease (MASLD) have been established in adult males; however, data on paediatric populations remain scarce. This study aimed to elucidate the sex-specific correlations of serum uric acid (SUA), alanine aminotransferase (ALT), and aspartate aminotransferase (AST) levels with MASLD in children with obesity. **Methods:** Clinical data from 262 children with obesity were retrospectively analysed. Participants were categorised by sex and MASLD status (MASLD+ vs. MASLD-). Laboratory tests, including ALT, AST, SUA, fasting glucose, glycated haemoglobin, lipid profile, and insulin levels, were compared. Comparison of significant influencing factors of obesity in children with non-alcoholic fatty liver disease was conducted using multivariable logistic regression analysis by sex. **Results:** Hyperinsulinemia was significantly associated with MASLD in all children. Sex-based analyses revealed differentiated patterns. In males, elevated SUA (*p* = 0.008) and ALT (*p* < 0.05) were independently associated with MASLD; however, in females, only elevated AST (*p* = 0.003) was significantly associated. **Conclusions:** While insulin resistance is a common risk factor for MASLD in all children with obesity, this study suggests that its manifestation may differ by sex. Elevated SUA and ALT levels may serve as sex-specific indicators in males, while elevated AST levels appear more relevant in females. These findings support the potential value of sex-specific metabolic markers in early MASLD screening, although further validation is needed.

## 1. Introduction

Non-alcoholic fatty liver disease (NAFLD), a chronic liver disorder characterised by excessive hepatic fat accumulation in the absence of excessive alcohol intake, is closely associated with obesity [1]. A recent consensus led by the American Association for the Study of Liver Diseases has officially renamed NAFLD as metabolic dysfunction-associated steatotic liver disease (MASLD). The new diagnostic criteria require evidence of hepatic steatosis, at least one cardiovascular metabolic risk factor, and the exclusion of other causes of fatty liver [2]. More than 98% of patients initially diagnosed with NAFLD meet the criteria for MASLD. This indicates a high degree of overlap and consistent clinical characteristics [3,4]. Therefore, this study followed the 2024 European Society Joint Guidelines [5] and adopted the MASLD terminology.

The incidence of MASLD has increased globally, particularly among children and adolescents. Moreover, the age of onset is becoming progressively younger [6,7]. In children, obesity, a risk factor for MASLD, is classified based on body mass index (BMI) as follows: overweight (BMI ≥ 85th percentile for age and sex), obesity (BMI ≥ 95th percentile), and severe obesity (BMI ≥ 120% of the 95th percentile or ≥ 35 kg/m^2^) [8]. Childhood obesity directly leads to liver steatosis and significantly increases the risk of cardiovascular diseases and diabetes in adulthood [9,10]. Children with obesity who develop MASLD also have a substantially higher risk of metabolic abnormalities. Previous studies have reported odds ratios ranging from 1.77 to 6.23 [11]. Currently, MASLD accounts for 15.8% of all reported causes of liver transplantation, posing a significant public health challenge [12].

Sex differences influence the development and progression of MASLD. Low testosterone levels in males lead to visceral fat accumulation and insulin resistance. In females, high androgen levels cause liver steatosis and other metabolic disorders, while oestrogen deficiency promotes central obesity and the development of metabolic syndrome [13]. The prevalence of MASLD is significantly higher in males than in females, both in adults and paediatric populations [1,14,15,16,17]. This difference is also evident in children aged 4–8 years. For example, a study on a cohort of Chinese children with obesity found that boys exhibited a higher incidence of MASLD before puberty than girls, which the authors attributed to sex-hormone-regulated differences in fat distribution [18]. Clinical studies have also demonstrated that males with MASLD often exhibit more pronounced markers of liver injury and metabolic disturbances, particularly elevated alanine aminotransferase (ALT) and serum uric acid (SUA) levels [17,19]. In a study of Chinese adults, Yang et al. [17] observed that simultaneous increases in ALT and SUA levels were more frequently observed in males, indicating possible sex-specific pathological differences in mechanisms. However, this conclusion is primarily based on the Chinese population, and its global application requires validation.

Current research on the mechanisms underlying sex differences in MASLD among the paediatric population remains limited, especially regarding the interaction between hepatic enzyme profiles and SUA in boys and girls affected by obesity, which has not been systematically elucidated. Therefore, this study focused on children with obesity to investigate the association between these differences and the incidence rate in males. By comparing the hepatic enzyme profiles and SUA levels between males and females with MASLD, the findings of this study will provide a theoretical basis for early screening and stratified intervention.

## 2. Materials and Methods

### 2.1. Study Participants

This retrospective study included children with obesity who met the criteria set by the 2018 Chinese ‘Screening for Overweight and Obesity among School-Age Children and Adolescents’ standard [20]. Between August 2018 and May 2022, our paediatric outpatient department treated and examined 262 children with obesity aged 6–13.5 years.

The diagnostic criteria for MASLD were established by expert consensus in 2023. All patients underwent abdominal ultrasound, which revealed increased liver echogenicity and posterior acoustic attenuation consistent with fatty liver disease. Obesity has been linked to cardiovascular disease. The exclusion criteria included concurrent diseases related to obesity, such as diabetes and cardiovascular disease; major comorbidities, such as kidney disease, mental disorder, and genetic obesity; and other liver diseases caused by known reasons, such as viral hepatitis, autoimmune liver disease, and drug-induced liver injury.

The study protocol was approved by the Ethics Committee at Beijing Jishuitan Hospital, Capital Medical University. The study adheres to the STROBE standard reporting guidelines. All participating children and their parents were informed of the study’s purpose, and written consent was obtained.

### 2.2. Data Processing

#### 2.2.1. Patient Characteristics

During the study, we recorded each participant’s clinical examination results and medical history and organised the data using standardised forms. The physical examination was conducted by paediatric endocrinologists who had received uniform training, aimed at assessing pubertal development, including the evaluation of breast development in females and testicular volume in males. To ensure measurement accuracy, height and weight were assessed using specially designed standing scales, achieving a precision of 0.1 cm and 0.1 kg, respectively. These rigorous data collection methods ensured the scientific rigour of the research and provided a reliable foundation for subsequent analysis. BMI was calculated using the following formula: weight (kg)/height squared (m^2^). BMI, height, and weight were standardised based on the standard deviations of Chinese children of the same sex and age, with results presented as the median [21,22].

#### 2.2.2. Laboratory Tests

We conducted a 10 h fasting observation on a group of children to study relevant indicators of paediatric MASLD. These children strictly refrained from eating and drinking from 21:00 until blood samples were collected the following morning. In the early morning, venipuncture was performed to measure ALT, aspartate aminotransferase (AST), SUA, fasting glucose, glycated haemoglobin, lipid profile, and insulin levels. ALT, AST, SUA, and lipids were analysed using a Hitachi 7600 fully automatic biochemical analyser (Hitachi High-Technologies, Tokyo, Japan); glycated haemoglobin was measured using a TOSOH G8 instrument (Tosoh Corporation, Yamaguchi-ken, Japan); and insulin levels were measured using a Roche E602 instrument (F.Hoffmann-La Roche Ltd., Basel, Switzerland). Additionally, according to the 2018 Chinese Paediatric NAFLD Diagnosis and Treatment Guidelines, professional sonographers conducted liver ultrasound examinations on the children to detect hepatic steatosis and rule out alcohol history and other specific aetiologies [23]. Ultimately, MASLD diagnosis was established.

Body fat percentage was measured using bioelectrical impedance analysis (BIA) with the Sihai Huachen H-Key350 eight-electrode BIA device (Shaanxi Sihai Huachen Technology Co., Ltd., Xi’an, China). Participants who fasted for 2.5 to 3 h before measurement were asked to empty their bladders, remove all metal items and jewellery, and wear light clothing. During the measurement, they held both electrode handles simultaneously and stood barefoot on the measuring platform, ensuring full contact with both hands and feet.

#### 2.2.3. Data Analysis

Data analysis was performed using SPSSPRO (Zhongyan Technology Co., Shanghai, China). Categorical variables were analysed using proportions and percentages, and differences were assessed using the Chi-square or Fisher’s exact tests. The Shapiro–Wilk test was used to assess the normality of quantitative variables. For normal distributions, means ± standard deviations and independent *t*-tests were used; for non-normal distributions, medians, interquartile ranges, and Mann–Whitney U tests were applied.

Logistic regression models were applied with distinctions made based on sex to analyse the relationship between ALT, AST, SUA, and MASLD among children with obesity. The level of statistical significance was set at *p* < 0.05.

## 3. Results

### 3.1. General Data Characteristics

A total of 262 children with obesity were included in the study (148 males and 114 females), with a mean age of 9.39 ± 1.82 years (range: 6–13.5 years). They were initially grouped by sex. Subsequently, based on abdominal ultrasound diagnosis, they were categorised into two groups: MASLD+ (*n* = 69, male:female = 52:17) and MASLD− (*n* = 193, male:female = 96:97). The incidence of MASLD among male and female children was 35.14% and 17.53%, respectively. The incidence rate in males was significantly higher than that in females (*p* < 0.001). In male children with obesity, the children in the MASLD+ group were significantly older, had greater body weight, higher BMI, and a higher percentage of body fat than those in the MASLD− group (Table 1). Conversely, in female children with obesity, no significant difference was observed in age (Table 1). However, the children in the MASLD+ group had significantly greater body weight, BMI, waist-to-hip ratio (WHR), and percentage of body fat than those in the MASLD− group. The male and female groups were further stratified based on pubertal status. There was no significant difference in the distribution of pre-pubertal and pubertal stages between the MASLD+ and MASLD− groups.

### 3.2. Laboratory Test Results

Table 2 presents the laboratory test results in males and females. Among male children with obesity, those in the MASLD+ group had significantly higher levels of insulin, ALT, AST, SUA, and high-density lipoprotein (HDL) than those in the MASLD− group. Among female children with obesity, those in the MASLD+ group had significantly higher levels of insulin, ALT, AST, and HDL than those in the MASLD− group; however, there was no significant difference in the SUA levels.

### 3.3. Sex-Stratified Multivariable Analysis of Risk Factors of MASLD

We first formally tested for effect modification by sex by including interaction terms (sex*variable) in the multivariable logistic regression model. As shown in Table S1, the interaction between sex and BMI was statistically significant (*p* < 0.05). However, the interactions between sex and other biomarkers (SUA, ALT, AST) did not reach statistical significance.

Although the study had limited statistical power to detect significant interactions due to the sample size, we conducted exploratory sex-stratified analyses to investigate heterogeneous patterns of association and to generate hypotheses for future research. The results of the sex-stratified multivariable analysis are presented in Table 3. Age, weight standard deviation score, BMI, percentage of body fat, pubertal stage (pre-puberty vs. puberty), insulin, ALT, AST, SUA, and HDL were included as independent variables, with the presence of MASLD as the dependent variable, in the logistic regression analysis. Insulin levels were significantly correlated with MASLD in all children with obesity (*p* < 0.05). Regarding sex differences, MASLD in males with obesity was significantly associated with elevated ALT and SUA levels (*p* < 0.05), whereas in females, it was significantly associated with elevated AST levels (*p* < 0.05).

## 4. Discussion

In this study, we observed that male children with obesity exhibited a significantly higher incidence of MASLD than female children with obesity, a finding consistent with the results from other cities in China [24]. An epidemiological study of MASLD among children and adolescents in Shanghai, China, reported a steady annual increase in incidence, rising from 2.1% in 2014 to 7.4% in 2020. However, the incidence rate among male children has consistently remained higher than that among female children [25]. Exploring the mechanisms underlying this significant sex difference in incidence will provide insights for potential therapeutic directions for reducing the incidence of MASLD in male children.

Herein, elevated insulin was a common and key factor in the development of MASLD in children with obesity, aligning closely with conclusions from previous research [26,27,28]. Insulin, a key peptide hormone secreted by pancreatic beta cells, plays a crucial regulatory role in maintaining whole-body glucose homeostasis and cellular survival by inhibiting BAX-mediated apoptosis and modulating mTOR-dependent autophagy [29]. The core mechanism underlying the onset of MASLD is insulin resistance and hyperinsulinemia, which can stimulate triglyceride synthesis in the liver [30].

Our sex-stratified analyses revealed differing patterns of association between hepatic enzyme profiles and SUA. Elevated serum ALT and uric acid levels were associated with MASLD in male children with obesity, whereas elevated serum AST levels were more significantly associated with MASLD in female children with obesity. This observed pattern suggests that MASLD in children with obesity may exhibit distinct metabolic characteristics depending on sex. However, it is important to note that the formal statistical tests for interactions between sex and these biomarkers (ALT, AST, SUA) were not statistically significant in our models (Table S1). Therefore, these findings should be interpreted as hypothesis-generating, suggesting potential sex-specific pathways rather than providing definitive evidence of effect modification by sex.

The elevation of ALT, one of the most sensitive serum biochemical indicators of liver cell damage, indicates a disruption of liver cell membrane integrity. This suggests that various damaging factors, such as oxidative stress and inflammatory responses, have stimulated liver cells, leading to the release of intracellular enzymes into the bloodstream [31]. The 2017 clinical practice guidelines from the North American Society for Pediatric Gastroenterology, Hepatology and Nutrition (NASPGHAN) recommend using the following sex-specific cutoff values for screening metabolic-associated fatty liver disease: ALT ≥ 2 times the upper limit of normal, defined as >52 IU/L for males and >44 IU/L for females [1]. When ALT levels exceed 80 IU/L, the incidence of metabolic-associated steatohepatitis in children reaches up to 41%, compared to 21% in those with ALT levels ≤ 80 IU/L [32]. A German cohort study further confirmed that the average ALT levels in healthy boys were significantly higher than those in girls of the same age, ranging from 11 months to 16 years, indicating that ALT is significantly influenced by sex [33]. In male children with obesity, elevated ALT levels may be associated with more significant visceral fat accumulation, stronger liver oxidative stress, and heightened inflammatory responses. Male children tend to deposit fat in abdominal and mesenteric visceral areas before and during puberty, while female children primarily accumulate subcutaneous fat [34]. High visceral fat activity readily breaks down into free fatty acids (FFAs), which are directly transported to the liver via the portal vein system, leading to excessive lipid accumulation in the liver and exacerbating fatty degeneration of liver cells [35]. FFAs released from visceral fat are oxidised in the liver, promoting the generation of reactive oxygen species. These induce lipid peroxidation reactions, activate hepatic macrophages and stellate cells, and stimulate the release of inflammatory factors, such as tumour necrosis factor-α and interleukin-6, as well as fibrosis-related factors [36]. This process damages liver cells and induces the release of ALT. Oestrogen may partially counteract this effect by enhancing adiponectin secretion and inhibiting liver fat production [37,38]. Therefore, the advantage-driven visceral fat accumulation may drive the portal vein FFA transport-liver oxidative stress/inflammation cascade, which could be the core pathological mechanism leading to significantly elevated ALT levels and a higher incidence of MASLD in boys with obesity compared to girls with obesity.

AST is present in the liver, heart muscle, skeletal muscle, kidneys, and the brain. Elevated AST levels in the presence of normal ALT levels may indicate cardiac or muscular disease [39]. In the liver, AST is primarily distributed in the mitochondria of hepatocytes, with a smaller amount in the cytoplasm. When pathological factors cause hepatocyte degeneration and increased cell membrane permeability, ALT is predominantly released. However, in cases of severe hepatocyte damage or necrosis, mitochondrial AST is released, leading to a significant increase in serum AST levels [40]. An elevated AST level often indicates more severe hepatocyte damage or progression of fibrosis [41,42]. In females with MASLD, oestrogen’s antioxidant effects may help delay the progression of fatty liver changes. However, once insulin resistance occurs, mitochondrial dysfunction and oxidative stress may become more pronounced, enhancing the release of AST [18]. This phenomenon may be related to greater mitochondrial sensitivity to metabolic stress in females, as well as sex-specific differences in the progression patterns of fibrosis.

High SUA levels are indicators of MASLD and play a role in its pathogenesis [43]. SUA contributes to the development and progression of MASLD through oxidative stress, inflammatory response, lipid metabolism disorders, and IR [44]. Xanthine oxidase inhibitors, which reduce uric acid levels, have been shown to provide significant benefits in the management of MASLD [45,46,47,48]. A survey study conducted on 184,342,210 individuals aged 12–80 years in the United States revealed that males had significantly higher SUA levels than females, with this difference emerging as early as puberty [49]. This may help explain why the incidence of MASLD is significantly higher in male than in female children with obesity.

This study has some limitations. It was a cross-sectional investigation that exclusively enrolled Chinese children with obesity aged 6–13.5 years, living in urban Beijing, and excluded other ethnic and geographic groups. Due to the small sample size, further stratified analysis based on Tanner staging was not conducted, resulting in a lack of specific studies on pubertal development stages. Currently, international sex-stratified analysis involving ALT, AST, and SUA for East Asian children is lacking; therefore, whether the findings of this study apply to non-Chinese populations remains to be verified. The PNPLA3-148M allele is more strongly associated with ALT levels in Hispanic populations, suggesting that genetic backgrounds influence the association patterns between metabolic markers and MASLD [50]. Another limitation is that the formal interaction tests between sex and biomarkers, such as SUA, ALT, and AST, did not reach statistical significance. Therefore, the differences observed in the stratified analyses should be considered as indicative patterns rather than conclusive evidence of sex-based effect modification. Moreover, the sample size of this study may have limited the statistical power to detect significant interactions. Future research should involve multicentre, large-scale studies to further explore the interaction effects between sex and key metabolic indicators (such as ALT and AST), pubertal stages, and BMI, particularly focusing on the heterogeneity of these interactions in childhood obesity among different ethnic groups. Based on this, precise diagnostic thresholds that integrate race, sex, pubertal stage, and BMI trajectories should be established to optimise early intervention strategies.

## 5. Conclusions

This study confirms hyperinsulinemia as a common critical risk factor for MASLD in all children with obesity. Furthermore, our exploratory sex-stratified analyses revealed differing patterns of association: SUA and ALT were associated with MASLD in males, while AST was associated with MASLD in females. Although the results of formal interaction tests were not significant for these markers, the observed patterns highlight the potential importance of a sex-aware approach in clinical assessment. Future studies with larger sample sizes are warranted to confirm these potential sex-specific associations. When evaluating children with obesity for MASLD, clinicians should consider not only universal markers, such as insulin, but also potentially sex-specific markers such as SUA, ALT, and AST.

## Figures and Tables

**Table 1:** General characteristics of children with obesity and MASLD.

Variable	Males (*n* = 148)			Females (*n* = 114)		
	MASLD+ (*n* = 52)	MASLD− (*n* = 96)	*p*	MASLD+ (*n* = 17)	MASLD− (*n* = 97)	*p*
Age (years) $\overset{-}{\times }$ ± s	10.23 ± 1.79	9.45 ± 1.89	0.016	9.53 ± 1.69	8.85 ± 1.62	0.137
Pre-puberty:puberty	24:28	56:40	0.158	3:14	25:72	0.140
Height SDS	1.95	1.48	0.744	1.57	1.32	0.399
M (p25, p75)	(0.69, 2.51)	(0.72, 2.48)		(1.07, 2.68)	(0.59, 2.36)	
Weight SDS	4.04	3.01	0.018	5.24 (3.75, 6.21)	3.27 (2.37, 4.80)	0.025
M (p25, p75)	(2.96, 5.23)	(2.13, 4.26)				
BMI z-score	2.78	2.49	0.013	3.55	2.60	0.006
M (P25, P75)	(2.38, 3.30)	(2.10, 3.04)		(2.84, 3.82)	(2.33, 3.14)	
WHR	0.87	0.84	0.073	0.86	0.80	0.016
M (P25, P75)	(0.82, 0.92)	(0.79, 0.89)		(0.81, 0.91)	(0.77, 0.85)	
Percentage of body fat (%)	40.90	37.30	0.001	39.60	33.65	0.008
M (P25, P75)	(36.70, 45.50)	(32.80, 40.76)		(34.10, 42.15)	(31.0, 38.63)	

MASLD, non-alcoholic fatty liver disease; MASLD−, children without MASLD; MASLD+, children with MASLD; WHR, waist-to-hip ratio; BMI z-score, body mass index z-score; SDS, standard deviation score; M, Median; P25, the 25th percentile; P75, the 75th percentile.

**Table 2:** Laboratory results of children with obesity and MASLD.

Variable	Males			Females		
	MASLD+	MASLD−	*p*	MASLD+	MASLD−	*p*
Vit D (ng/mL)	17.90	21.70	0.129	21.50	19.80	0.616
M (p25, p75)	(13.40, 25.10)	(15.98, 26.00)		(15.45, 25.45)	(13.65, 24.90)	
Glycated haemoglobin (%)	5.50	5.50	0.193	5.40	5.40	0.182
M (p25, p75)	(5.30, 5.60)	(5.30, 5.60)		(5.30, 5.70)	(5.20, 5.65)	
Insulin (μU/mL)	29.60	16.60	<0.001	44.80	16.20	<0.001
M (p25, p75)	(17.72, 35.58)	(11.65, 25.20)		(15.00, 53.60)	(11.00, 25.10)	
ALT (IU/L)	28.50	19.00	<0.001	29.00	20.00	<0.001
M (P25, P75)	(21.25, 44.75)	(16.00, 25.00)		(21.50, 33.50)	(16.00, 24.00)	
AST (IU/L)	25.50	22.00	<0.001	23.00	20.00	<0.001
M (P25, P75)	(22.00, 32.75)	(19.00, 26.00)		(21.50, 33.50)	(16.00, 24.00)	
GLU (mmol/L)	4.90	5.10	0160	4.90	5.00	0.260
M (P25, P75)	(4.70, 5.28)	(4.80, 5.30)		(4.80, 5.30)	(4.75, 5.20)	
SUA (μmol/L)	411.00	354.00	<0.001	343.00	349.00	0.102
M (P25, P75)	(364.25, 462.00)	(297.00, 396.50)		(313.50, 448.50)	(302.50, 396.00)	
CHO (mmol/L)	4.50	4.42	0.452	4.27	4.40	0.566
M (P25, P75)	(3.90, 4.92)	(4.04, 5.04)		(4.00, 4.54)	(3.94, 5.08)	
TGs (mmol/L)	1.27	1.06	0.060	1.23	1.03	0.426
M (P25, P75)	(0.85, 1.79)	(0.77, 1.47)		(0.86, 1.58)	(0.68, 1.45)	
HDL (mmol/L)	1.31	0.73	0.002	1.26	1.39	0.002
M (P25, P75)	(1.14, 1.48)	(0.46, 1.12)		(1.12, 1.32)	(1.24, 1.59)	
LDL (mmol/L)	2.63	2.55	0.840	2.51	2.51	0.628
M (P25, P75)	(2.09, 2.99)	(2.10, 3.06)		(2.38, 2.80)	(2.09, 3.14)	
ALB (g/L)	46.90	46.40	0.823	46.40	46.70	0.800
M (P25, P75)	(45.80, 48.47)	(44.95, 48.20)		(44.95, 48.55)	(45.10, 48.30)	

MASLD, non-alcoholic fatty liver disease; MASLD−, children without MASLD; MASLD+, children with MASLD; Vit D, Vitamin D; AST, aspartate transaminase; ALT, alanine aminotransferase; GLU, glucose; SUA, serum uric acid; TGs, triglycerides; CHO, cholesterol; LDL, low-density lipoproteins; HDL, high-density lipoproteins; ALB, albumin; M, Median; P25, the 25th percentile; P75, the 75th percentile.

**Table 3:** Multifactorial analysis of MASLD in children of different sexes with obesity.

Variable	Males				Females			
	β	*P*	OR	95% CI	Β	*p*	OR	95% CI
ALT	0.072	<0.001	1.075	1.032–1.119	0.065	0.169	1.072	0.969–1.170
AST	0.047	0.289	1.048	0.961–1.142	0.146	0.003	1.157	1.051–1.274
SUA	0.008	0.008	1.008	1.002–1.013	−0.006	0.251	0.994	0.984–1.004
Insulin	0.053	0.003	1.055	1.018–1.092	0.038	0.004	1.039	1.013–1.066
BMI z-score	−0.198	0.854	0.821	0.100–6.748	2.459	0.111	11.697	0.571–239.801
Age	0.121	0.598	1.129	0.720–1.771	0.349	0.386	1.417	0.644–3.120
Weight SDS	0.359	0.466	1.431	0.545–3.758	−0.408	0.447	0.665	0.232–1.902
Height SDS	−0.395	0.292	0.674	0.323–1.404	0.432	0.410	1.541	0.551–4.311
Percentage of body fat	0.032	0.490	1.033	0.943–1.131	−0.093	0.424	0.911	0.725–1.145
HDL	−1.251	0.245	0.283	0.034–2.371	−1.694	0.322	0.184	0.006–5.242
Pubertal stage	0.166	0.766	1.181	0.395–3.536	1.576	0.190	4.834	0.458–51.064

SUA, serum uric acid; AST, aspartate transaminase; ALT, alanine aminotransferase; OR, odds ratio; CI, confidence interval; BMI z-score, body mass index z-score; SDS, standard deviation score; HDL, high-density lipoprotein.

## Data Availability

Due to privacy concerns, the datasets referenced in this article are not publicly available. Requests for access may be directed to tianbing1214@sina.com.

## Supplementary Materials

The following supporting information can be downloaded at: https://www.mdpi.com/article/10.3390/healthcare13172219/s1, Table S1:Multivariable logistic regression with interaction test between sex and other variables with Obesity and MASLD.

## Abbreviations

The following abbreviations are used in this manuscript:

WHR	waist-to-hip ratio
BMI z-score	body mass index z-score
NAFLD	non-alcoholic fatty liver disease
MASLD	metabolic dysfunction-associated steatotic liver disease
MASLD−	children without MASLD
MASLD+	children with MASLD
Vit D	vitamin D
AST	aspartate transaminase
ALT	alanine aminotransferase
GLU	glucose
SUA	serum uric acid
CHO	cholesterol
LDL	low-density lipoprotein
HDL	high-density lipoprotein

## References

[1] Vos M.B., Abrams S.H., Barlow S.E., Caprio S., Daniels S.R., Kohli R., Mouzaki M., Sathya P., Schwimmer J.B., Sundaram S.S. (2017). NASPGHAN clinical practice guideline for the diagnosis and treatment of nonalcoholic fatty liver disease in children: Recommendations from the Expert Committee on NAFLD (ECON) and the North American Society of Pediatric Gastroenterology, Hepatology and Nutrition (NASPGHAN). J. Pediatr. Gastroenterol. Nutr..

[2] Rinella M.E., Lazarus J.V., Ratziu V., Francque S.M., Sanyal A.J., Kanwal F., Romero D., Abdelmalek M.F., Anstee Q.M., Arab J.P. (2023). A multisociety Delphi consensus statement on new fatty liver disease nomenclature. J. Hepatol..

[3] Younossi Z.M., Paik J.M., Stepanova M., Ong J., Alqahtani S., Henry L. (2024). Clinical profiles and mortality rates are similar for metabolic dysfunction-associated steatotic liver disease and non-alcoholic fatty liver disease. J. Hepatol..

[4] Hardy T., Wonders K., Younes R., Aithal G.P., Aller R., Allison M., Bedossa P., Betsou F., Boursier J., Brosnan M.J. (2020). The European NAFLD Registry: A real-world longitudinal cohort study of nonalcoholic fatty liver disease. Contemp. Clin. Trials..

[5] European Association for the Study of the Liver (EASL), European Association for the Study of Diabetes (EASD), European Association for the Study of Obesity (EASO) (2024). EASL-EASD-EASO Clinical Practice Guidelines on the management of metabolic dysfunction-associated steatotic liver disease (MASLD). J. Hepatol..

[6] Clemente M.G., Mandato C., Poeta M., Vajro P. (2016). Pediatric non-alcoholic fatty liver disease: Recent solutions, unresolved issues, and future research directions. World J. Gastroenterol..

[7] Bonsembiante L., Targher G., Maffeis C. (2022). Non-alcoholic fatty liver disease in obese children and adolescents: A role for nutrition?. Eur. J. Clin. Nutr..

[8] Kelly A.S., Barlow S.E., Rao G.R., Inge T.H., Hayman L.L., Steinberger J., Urbina E.M., Ewing L.J., Daniels S.R., American Heart Association Atherosclerosis (2013). Severe obesity in children and adolescents: Identification, associated health risks, and treatment approaches: A scientific statement from the American Heart Association. Circulation.

[9] Field A.E., Cook N.R., Gillman M.W. (2005). Weight status in childhood as a predictor of becoming overweight or hypertensive in early adulthood. Obes. Res..

[10] Bellini M.I., Urciuoli I., Del Gaudio G., Polti G., Iannetti G., Gangitano E., Lori E., Lubrano C., Cantisani V., Sorrenti S. (2022). Nonalcoholic fatty liver disease and diabetes. World J. Diabetes.

[11] Ye P., Gao L., Xia Z., Peng L., Shi X., Ma J., Dong Y., Dai D., Yang Q., Chen X. (2024). Association between non-alcoholic fatty liver disease and metabolic abnormalities in children with different weight statuses. Public Health.

[12] Wong R.J., Aguilar M., Cheung R., Perumpail R.B., Harrison S.A., Younossi Z.M., Ahmed A. (2015). Nonalcoholic steatohepatitis is the second leading etiology of liver disease among adults awaiting liver transplantation in the United States. Gastroenterology.

[13] Gangitano E., Scannapieco F., Lubrano C., Gnessi L. (2024). Metabolic syndrome, hepatic steatosis and testosterone: A matter of sex. Livers.

[14] Wiegand S., Keller K.M., Röbl M., L’Allemand D., Reinehr T., Widhalm K., Holl R.W., APV-Study Group and the German Competence Network Adipositas (2010). Obese boys at increased risk for nonalcoholic liver disease: Evaluation of 16,390 overweight or obese children and adolescents. Int. J. Obes..

[15] Malespin M., Sleesman B., Lau A., Wong S.S., Cotler S.J. (2015). Prevalence and correlates of suspected nonalcoholic fatty liver disease in Chinese American children. J. Clin. Gastroenterol..

[16] Zelber-Sagi S., Ben-Assuli O., Rabinowich L., Goldstein A., Magid A., Shalev V., Shibolet O., Chodick G. (2015). The association between the serum levels of uric acid and alanine aminotransferase in a population-based cohort. Liver Int..

[17] Yang H., Li D., Song X., Liu F., Wang X., Ma Q., Zhang X., Li X. (2018). Joint associations of serum uric acid and ALT with NAFLD in elderly men and women: A Chinese cross-sectional study. J. Transl. Med..

[18] Jin B., Wu Z., Wang S., Yu Z., Ullah R., Liang X., Wu W., Huang K., Ni Y., Wang J. (2024). Gender differences in non-alcoholic fatty liver disease in obese children and adolescents: A large cross-sectional study. Hepatol. Int..

[19] Luo J., Liu L.W., Liu J.M., Shi Y.W., Sun Y.M., Wang Q.Y., Wang M., Fan X., Ou X.J., Zhao X.Y. (2021). Comparative study of clinicopathological features, and risk factors of advanced fibrosis between genders with non-alcoholic fatty liver disease. Chin. J. Hepatol..

[20] (2018). Screening for Overweight and Obesity in School-Age Children and Adolescents.

[21] Li H., Ji C.Y., Zong X.N., Zhang Y.Q. (2009). Body mass index growth curves for Chinese children and adolescents aged 0 to 18 years. Zhonghua Er Ke Za Zhi.

[22] Li H., Ji C.Y., Zong X.N., Zhang Y.Q. (2009). Height and weight standardized growth charts for Chinese children and adolescents aged 0 to 18 years. Zhonghua Er Ke Za Zhi.

[23] Endocrinology, Genetics and Metabolism Group, Pediatrics Society of Chinese Medical Association, Gastroenterology Group, Pediatrics Society of Chinese Medical Association, Adolescent Medicine Specialty Committee, Pediatrics Society of Chinese Medical Association (2018). Experts consensus on diagnosis and treatment of nonalcoholic fatty liver disease in children. Chin. J. Practical. Pediatr..

[24] Wang J., Lin H., Chiavaroli V., Jin B., Yuan J., Huang K., Wu W., Dong G., Derraik J.G.B., Fu J. (2022). High prevalence of cardiometabolic comorbidities among children and adolescents with severe obesity from a large metropolitan centre (Hangzhou, China). Front. Endocrinol..

[25] Chen X., Wen X., Zhang Y., Zhu X., Dou Y., Han Y., Wang Y., Hu Y.H., He W., Chen H. (2022). A cross-sectional survey of the 7-year prevalence of non-alcoholic fatty liver disease among children and adolescents in Minhang District, Shanghai. Chin J. Evid. Based. Pediatr..

[26] Furthner D., Anderwald C.-H., Bergsten P., Forslund A., Kullberg J., Ahlström H., Manell H., Ciba I., Mangge H., Maruszczak K. (2022). Single Point Insulin Sensitivity Estimator in Pediatric Non-Alcoholic Fatty Liver Disease. Front. Endocrinol.

[27] Yetim A., Şahin M., Kandemir L., Bulakçı B., Aksakal M.T., Karapınar E., Sever H., Baş F. (2024). Evaluation of the ability of insulin resistance and lipid-related indices to predict the presence of NAFLD in obese adolescents. Lipids Health Dis..

[28] Franceschi R., Fintini D., Ravà L., Mariani M., Aureli A., Inzaghi E., Pedicelli S., Deodati A., Bizzarri C., Cappa M. (2023). Insulin Clearance at the Pubertal Transition in Youth with Obesity and Steatosis Liver Disease. Int. J. Mol. Sci..

[29] Sharma M.D., Garber A.J., Farmer J.A. (2008). Role of insulin signaling in maintaining energy homeostasis. Endocr. Pract..

[30] Palma R., Pronio A., Romeo M., Scognamiglio F., Ventriglia L., Ormando V.M., Lamazza A., Pontone S., Federico A., Dallio M. (2022). The role of insulin resistance in fueling NAFLD pathogenesis: From molecular mechanisms to clinical implications. J. Clin. Med..

[31] Thakur S., Kumar V., Das R., Sharma V., Mehta D.K. (2024). Biomarkers of Hepatic Toxicity: An Overview. Curr. Ther. Res. Clin. Exp..

[32] Schwimmer J.B., Newton K.P., Awai H.I., Choi L.J., Garcia M.A., Ellis L.L., Vanderwall K., Fontanesi J. (2013). Paediatric gastroenterology evaluation of overweight and obese children referred from primary care for suspected non-alcoholic fatty liver disease. Aliment. Pharmacol. Ther..

[33] Bussler S., Vogel M., Pietzner D., Harms K., Buzek T., Penke M., Händel N., Körner A., Baumann U., Kiess W. (2018). New pediatric percentiles of liver enzyme serum levels (alanine aminotransferase, aspartate aminotransferase, γ-glutamyltransferase): Effects of age, sex, body mass index, and pubertal stage. Hepatology.

[34] Staiano A.E., Katzmarzyk P.T. (2012). Ethnic and sex differences in body fat and visceral and subcutaneous adiposity in children and adolescents. Int. J. Obes..

[35] Mirza M.S. (2011). Obesity, visceral fat, and NAFLD: Querying the role of adipokines in the progression of nonalcoholic fatty liver disease. ISRN Gastroenterol..

[36] Tang S.P., Mao X.L., Chen Y.H., Yan L.L., Ye L.P., Li S.W. (2022). Reactive Oxygen Species Induce Fatty Liver and Ischemia-Reperfusion Injury by Promoting Inflammation and Cell Death. Front. Immunol..

[37] Palmisano B.T., Zhu L., Stafford J.M. (2017). Role of estrogens in the regulation of liver lipid metabolism. Adv. Exp. Med. Biol..

[38] DiStefano J.K. (2020). NAFLD and NASH in Postmenopausal women: Implications for diagnosis and treatment. Endocrinology.

[39] Kwo P.Y., Cohen S.M., Lim J.K. (2017). ACG Clinical Guideline: Evaluation of abnormal liver chemistries. Am. J. Gastroenterol..

[40] Li F., Lu L.G. (2015). Evaluation of abnormal liver function and its clinical significance. J. Clin. Hepatol..

[41] Yun H., Shi J., Hu C., Zhang L., Liu H., Lou G. (2010). Serum AST levels are associated with progressive hepatic fibrosis in non-alcoholic fatty liver disease. Chin. Hepatol..

[42] Gong X., Li S., Chen S., Wang M. (2016). Study on the correlation between ALT, AST, UA, and Non-alcoholic fatty liver disease pathology. J. Med. Res..

[43] Russo E., Leoncini G., Esposito P., Garibotto G., Pontremoli R., Viazzi F. (2020). Fructose and uric acid: Major mediators of cardiovascular disease risk starting at pediatric age. Int. J. Mol. Sci..

[44] Fan J., Wang D. (2024). Serum uric acid and nonalcoholic fatty liver disease. Front. Endocrinol..

[45] Xu C. (2016). Hyperuricemia and nonalcoholic fatty liver disease: From bedside to bench and back. Hepatol. Int..

[46] Sun D.Q., Wu S.J., Liu W.Y., Lu Q.D., Zhu G.Q., Shi K.Q., Braddock M., Song D., Zheng M.H. (2016). Serum uric acid: A new therapeutic target for nonalcoholic fatty liver disease. Expert. Opin. Ther. Targets..

[47] Barişik V., Korkmaz H.A., Çekdemir Y.E., Torlak D., Aktuğ H., Yavaşoğlu A., Erbaş O. (2023). The therapeutic effect of allopurinol in fatty liver disease in rats. Acta Endocrinol..

[48] Al-Shargi A., El Kholy A.A., Adel A., Hassany M., Shaheen S.M. (2023). Allopurinol versus febuxostat: A new approach for the management of hepatic steatosis in metabolic dysfunction-associated steatotic liver disease. Biomedicines.

[49] Wang Y., Charchar F.J. (2021). Establishment of sex difference in circulating uric acid is associated with higher testosterone and lower sex hormone-binding globulin in adolescent boys. Sci. Rep..

[50] Romeo S., Kozlitina J., Xing C., Pertsemlidis A., Cox D., Pennacchio L.A., Boerwinkle E., Cohen J.C., Hobbs H.H. (2008). Genetic variation in PNPLA3 confers susceptibility to nonalcoholic fatty liver disease. Nat. Genet..

