# Supplementary material for: Metabolic Dysfunction-Associated Steatotic Liver Disease in Children with Obesity: Sex-Stratified Analysis of Hepatic Enzyme Profiles and Serum Uric Acid
Source: Healthcare (Basel). 2025 Sep 4;13(17):2219. doi: 10.3390/healthcare13172219 (PMC12428106; doi:10.3390/healthcare13172219)
Supplement: Supplementary file 1 [file healthcare-13-02219-s001.zip › healthcare-3721294-supplementary.pdf]

Table S1. **Multivariable logistic regression with interaction test between sex and other variables with Obesity and MASLD**

Variable	β	SE	Wald	P	OR	95%CI	
						Lower	Upper
interactrions between SEX and pubertal stage	1.071	1.366	0.615	0.433	2.919	0.201	42.445
interactrions between SEX and ALB	-0.143	0.16	0.805	0.370	0.867	0.634	1.185
interactrions between SEX and LDL	0.396	0.922	0.185	0.667	1.486	0.244	9.048
interactrions between SEX and TG	0.289	0.719	0.162	0.687	1.335	0.326	5.465
interactrions between SEX and CHO	-0.615	0.878	0.49	0.484	0.541	0.097	3.024
interactrions between SEX and SUA	-0.005	0.006	0.722	0.395	0.995	0.983	1.007
interactrions between SEX and GLU	0.233	1.156	0.041	0.840	1.263	0.131	12.168
interactrions between SEX and AST	0.171	0.102	2.824	0.093	1.186	0.972	1.447
interactrions between SEX and ALT	0.081	0.056	2.107	0.147	1.084	0.972	1.209
interactrions between SEX and Insulin	0.028	0.021	1.837	0.175	1.029	0.987	1.072
interactrions between SEX and Glycated hemoglobin	-1.845	1.446	1.629	0.202	0.158	0.009	2.686
interactrions between SEX and Vit D	0.029	0.063	0.211	0.646	1.029	0.91	1.165
interactrions between SEX and WHR	-1.331	8.988	0.022	0.882	0.264	0	11818625.649
interactrions between SEX and percentage of body fat	-0.011	0.009	1.435	0.231	0.989	0.972	1.007
interactrions between SEX and BMIz-score	1.678	0.83	4.085	0.043**	5.354	1.052	27.25
interactrions between SEX and Weight SDS	0.011	0.01	1.177	0.278	1.011	0.991	1.03
interactrions between SEX and Height SDS	0.017	0.013	1.793	0.181	1.017	0.992	1.042
interactrions between SEX and Age	0.332	0.422	0.617	0.432	1.393	0.609	3.188

MASLD, non-alcoholic fatty liver disease; MASLD-, children without MASLD; MASLD+, children with MASLD; WHR, waist-to-hip ratio; BMI z-score, body mass index z-score; SDS, standard deviation score; Vit D, Vitamin D; AST, aspartate transaminase; ALT, alanine aminotransferase; GLU, glucose; SUA, serum uric acid; TGs, triglycerides; CHO, cholesterol; LDL, low-density lipoproteins; HDL, high-density lipoproteins; ALB, albumin.
